# Zerumbone suppresses IKKα, Akt, and FOXO1 activation, resulting in apoptosis of GBM 8401 cells

**DOI:** 10.1186/1423-0127-19-86

**Published:** 2012-10-05

**Authors:** Hsing-Yu Weng, Ming-Jen Hsu, Ching-Chung Wang, Bing-Chang Chen, Chuang-Ye Hong, Mei-Chieh Chen, Wen-Ta Chiu, Chien-Huang Lin

**Affiliations:** 1 Graduate Institute of Clinical Medicine, Taipei Medical University, No.250, Wu-Hsing Street, 11031, Taipei, Taiwan; 2 Department of Neurology, Wan Fang Hospital, Taipei Medical University, No.111, Sec. 3, Hsing-Long Road, Taipei, 11696, Taiwan; 3 Department of Pharmacology, College of Medicine, Taipei Medical University, No. 250, Wu-Hsing Street, 11031, Taipei, Taiwan; 4 Graduate Institute of Pharmacognosy, College of Pharmacy, Taipei Medical University, No. 250, Wu-Hsing Street, 11031, Taipei, Taiwan; 5 School of Respiratory therapy, College of Medicine, Taipei Medical University, No. 250, Wu-Hsing Street, 11031, Taipei, Taiwan; 6 School of Medicine, College of Medicine, Taipei Medical University, No. 250, Wu-Hsing Street, 11031, Taipei, Taiwan; 7 Graduate Institute of Medical Science, College of Medicine, Taipei Medical University, No.250, Wu-Hsing Street, 11031, Taipei, Taiwan

**Keywords:** Zerumbone, IKK, Akt, FOXO1, Glioblastoma multiforme

## Abstract

**Background:**

Zerumbone, a sesquiterpene compound isolated from subtropical ginger, *Zingiber zerumbet* Smith, has been documented to exert antitumoral and anti- inflammatory activities. In this study, we demonstrate that zerumbone induces apoptosis in human glioblastoma multiforme (GBM8401) cells and investigate the apoptotic mechanism.

**Methods:**

We added a caspase inhibitor and transfected wild-type (WT) IKK and Akt into GBM 8401 cells, and measured cell viability and apoptosis by MTT assay and flow cytometry. By western blotting, we evaluated activation of caspase-3, dephosphorylation of IKK, Akt, FOXO1 with time, and change of IKK, Akt, and FOXO1 phosphorylation after transfection of WT IKK and Akt.

**Results:**

Zerumbone (10∽50 μM) induced death of GBM8401 cells in a dose-dependent manner. Flow cytometry studies showed that zerumbone increased the percentage of apoptotic GBM cells. Zerumbone also caused caspase-3 activation and poly (ADP-ribose) polymerase (PARP) production. *N*-benzyloxycarbonyl -Val-Ala-Asp- fluoromethylketone (zVAD-fmk), a broad-spectrum caspase inhibitor, hindered zerumbone-induced cell death. Transfection of GBM 8401 cells with WT IKKα inhibited zerumbone-induced apoptosis, and zerumbone significantly decreased IKKα phosphorylation levels in a time-dependent manner. Similarly, transfection of GBM8401 cells with Akt suppressed zerumbone-induced apoptosis, and zerumbone also diminished Akt phosphorylation levels remarkably and time-dependently. Moreover, transfection of GBM8401 cells with WT IKKα reduced the zerumbone-induced decrease in Akt and FOXO1 phosphorylation. However, transfection with WT Akt decreased FOXO1, but not IKKα, phosphorylation.

**Conclusion:**

The results suggest that inactivation of IKKα, followed by Akt and FOXO1 phosphorylation and caspase-3 activation, contributes to zerumbone-induced GBM cell apoptosis.

## Background

Zerumbone (2,6,9,9- tetramethyl- [2*E*,6*E*,10*E*- cycloundeca- 2,6,10-trien- 1-one) is a sesquiterpenoid compound extracted from the rhizomes of wild ginger, *Zingiber zerumbet* Smith, which is widely distributed in Southeast Asia [1]. Several recent studies revealed that zerumbone can inhibit tumor initiation and proliferation. This compound inhibits the proliferation of colon [2,3] and breast cancers [3], with minimal effects on normal cells [2]. Zerumbone was also shown to suppress skin tumor initiation and promotion [4], inhibit inducible nitric oxide synthase (iNOS) and cyclooxygenase (COX)-2 expression, suppress free radical generation, and inhibit tumor necrosis factor (TNF)-α release in activated leukocytes. Moreover, zerumbone suppresses the activation of nuclear factor kappa- light- chain- enhancer of activated B cells (NF-κB) and NF-κB-related gene expression induced by carcinogens in several different kinds of cells [5].

NF-κB is a transcription factor that regulates various cellular processes such as cellular growth, development, immune and inflammatory responses, and apoptosis [6-8]. In most cells, NF-κB is retained in the cytoplasm because IκB proteins mask the nuclear localization sequence of NF-κB. Activated- IκB kinase (IKK) induces the phosphorylation and rapid ubiquitin-dependent degradation of IκB. The cytosolic NF-κB is then released and translocated to the nucleus, where it modifies gene transcription [9,10]. IKKs are formed by a high-molecular-weight complex containing at least 2 catalytic subunits, IKKα and IKKβ, and the associated regulatory subunit IKKγ (NEMO) [6,10,11]. In most circumstances, the IKKα and IKKβ kinases both have separate upstream signaling pathways and downstream targets [12,13]. The IKKβ kinase principally involves the innate immunity responses as well as cancer signals; however, IKKα regulates differentiation and growth responses [14].

Several studies have demonstrated that the phosphoinositide-3-OH-kinase (PI3K)/Akt pathway activates the NF-κB system [15,16]. PI3K is often involved in survival pathways stimulated by various growth factors, and it protects cells from apoptotic cell death [17,18]. Akt, a serine/threonine kinase, mediates many PI3K-regulated biological responses including glucose uptake, protein synthesis, and inhibition of apoptosis [18-21]. Overexpression of Akt, especially constitutively active Akt, protects cells against apoptosis, and even promotes malignant transformation, whereas inhibition of Akt activity stimulates apoptosis in certain mammalian cells [22]. Activated Akt can enhance cell survival by phosphorylating several downstream targets, including the Bcl-2 family member BAD (Bcl-2-associated death promoter), IΚΚ, caspase family member caspase-9, and the forkhead family transcription factor FKHRL1 [21,23-28].

Some studies reported that IKK can induce phosphorylation, ubiquitination, and degradation of forkhead box, class O (FOXO) factors, and promote cell proliferation and tumorigenesis [29]. Therefore, it is possible that the IKK pathway may be involved in regulating the transactivation activities of FOXO members. The FOXO factors, which include FKHR (FOXO1), FKHRL1 (FOXO3a) and AFX (FOXO4), share DNA-binding specificity to a core consensus site [30]. The FOXO members are downstream targets of PI3K/Akt signaling. Phosphorylation of the FOXO members by Akt inhibits their transcriptional activity. FOXO1 is phosphorylated on 3 sites (Thr-24, Ser-256, and Ser-319) in a PI3K-dependent manner [31], and phosphorylation on all or a subset of these sites contributes to the inactivation of its transcriptional activity [32].

In adults, glioblastoma multiforme (GBM) is the most common primary malignant brain tumor. The median survival duration of GBM patients is usually less than 1 year from the time of diagnosis [33,34]. The standard treatment for the tumor includes surgical resection to the maximal and safest extent, radiotherapy and systemic chemotherapy. Even with the most aggressive treatment and the most up-to-date chemotherapy, the median survival time is less than 15 months [35]. Therefore, it is necessary to continue the development of more effective chemotherapeutic agents to improve the survival rate of GBM patients.

In this study, we investigate the roles of IKK, Akt, and FOXO1 in zerumbone-induced apoptosis of human GBM8401 cells. Our data demonstrate that zerumbone induces GBM cell apoptosis, which is mediated by inactivation of IKK, followed by inactivation of Akt-FOXO1 cascade and activation of caspase-3.

## Methods

### Materials

Zerumbone was kindly provided by Dr. Ching-Chung Wang (Graduate Institute of Pharmacognosy, College of Pharmacy, Taipei Medical University, Taiwan). Dulbecco’s modified Eagle’s medium (DMEM), fetal calf serum (FCS), penicillin/streptomycin, OptiMEM, and Lipofectamine plus™ reagent were purchased from Invitrogen (Carlsbad, CA, USA). Antibodies specific for Bcl-2, Bax, Bcl-XL, Akt and procaspase-3 were purchased from Santa Cruz Biotechnology (Santa Cruz, CA, USA). Akt and horseradish peroxidase-conjugated anti-mouse and anti-rabbit antibodies were also purchased from Santa Cruz Biotechnology. Wild-type (WT)-IKKα and WT-IKKβ constructs were kindly provided by Dr. Michael Karin (Department of Pharmacology, School of Medicine, University of California-San Diego, San Diego, CA, USA). Antibodies specific for phospho-Akt (Ser473), phospho-IKK (Ser 180/181) and phospho-FOXO1 (ser 319) were purchased from Cell Signaling Technology (Beverly, MA, USA). The enhanced chemiluminescence detection agent was purchased from PerkinElmer Life Sciences (Boston, MA, USA). All materials for sodium dodecylsulfate polyacrylamide gel electrophoresis (SDS-PAGE) were obtained from Hoefer (Holliston, MA, USA). The pUSEamp-Akt1 complementary(c)DNA (WT-Akt) was purchased from Upstate Biotechnology (Lake Placid, NY, USA). Propidium iodide (PI), *N*- benzyloxycarbonyl- Val- Ala- Asp- fluoromethylketone (zVAD-fmk), dithiothreitol (DTT), phenylmethylsulphonyl fluoride (PMSF), pepstatin A, leupeptin, SDS, 3-(4,5-dimethyl-thiazol-2-yl) -2,5- diphenyltetrazolium (MTT), and other chemicals were obtained from Sigma (St. Louis, MO, USA).

### Cell culture

GBM8401 cells, kindly given by Professor Yen-Chou Chen (Graduate Institute of Medical Sciences, Taipei Medical University, Taiwan), and U87MG cells, obtained from the American Type Culture Collection, are both permanent human brain glioblastoma cell lines and were cultured in DMEM with 10% FCS and antibiotics (100 U/ml penicillin and 100 μg/ml streptomycin).

### Cell viability assay

Cell viability was measured by a previously described colorimetric MTT assay [20,36]. Briefly, cells (10^5^ cells/well) were cultured in 12-well plates and incubated with dimethyl sulfoxide (DMSO) or various concentrations (10 μM, 30 μM, or 50 μM) of zerumbone for 24 h. After various treatments, 5 mg/ml MTT was added to the culture plates and the plates were incubated at 37°C for an additional 4 h. The cells were then lysed in 500 μl of DMSO. The absorbance at 550 nm was measured on a microplate reader. Samples were plated and assayed in triplicate and the experiment was repeated at least 3 times.

### Flow cytometric analysis

GBM8401 cells were cultured in 10-cm Petri dishes. After reaching confluence, cells were treated with DMSO or 10 μM, 30 μM, or 50 μM of zerumbone for 24 h. After treatment, cells were harvested and washed twice with phosphate-buffered saline (PBS: 137 mM NaCl, 2.7 mM KCl, 4.3 mM Na_2_HPO_4_, and 1.5 mM KH_2_PO4; pH 7.4), and re-suspended in ice-cold 70% ethanol at -20°C overnight. Cells were washed for 5 min with 0.4 ml phosphate-citric acid buffer (pH 7.8) containing 50 mM Na_2_HPO_4_, 25 mM citric acid, and 0.1% Triton X-100 and subsequently stained with 1.5 ml PI staining buffer containing 0.5% Triton X-100, 10 mM PIPES, 100 mM NaCl, 2 mM MgCl_2_, 0.1 U/ml RNase A, and 25 μg/ml PI for 30 min in the dark before the flow cytometric analysis. Samples were analyzed by FACScan using the CellQuest software (Becton Dickinson, San Jose, CA, USA).

### Immunoblot analysis

To determine the levels of procaspase-3, PARP, Bcl-2, Bax, Bcl-XL, α-tubulin, phospho-Akt (Ser473), phospho-IKK(ser180/181), and phospho-FOXOI (ser319) in GBM8401 cells, the proteins were extracted as described previously [37], with modifications. Briefly, GBM8401 cells were cultured in 6-cm dishes. After the cells reached confluence, they were treated with DMSO or 50 μM zerumbone for different time periods. After incubation, cells were washed twice with ice-cold PBS and solubilized in extraction buffer containing 10 mM Tris (pH 7.0), 140 mM NaCl, 3 mM MgCl_2_, 2 mM PMSF, 5 mM DTT, 0.5% NP-40, 0.01 mg/ml aprotinin, 0.01 mg/ml leupeptin, 1 mM benzamidine, and 1 mM Na_3_VO_4_. Protein concentrations of thecell lysates were determined by the Bradford protein assay (Hoefer). An equal amount of protein (30 μg) in each sample was boiled in SDS sample loading buffer, and then fractionated on SDS-PAGE before blotting onto a polyvinylidene difluoride (PVDF) membrane. Blots were then incubated in 150 mM NaCl, 20 mM Tris, and 0.02% Tween (pH 7.4) containing 5% non-fat milk. Proteins were visualized by specific primary antibodies and then incubated with alkaline phosphatase- or horseradish peroxidase-conjugated second antibodies. After washing with PBS, blots were developed using NBT/BCIP or an enhanced chemiluminescence kit according to the manufacturer’s instructions before exposure to photographic films.

### Plasmid DNA transfection

GBM8401 cells were seeded at a density of 10^5^ cells/ml into 12-well plates. On the following days, cells were transfected with Lipofectamine plus™ reagent containing 1 μg/well of pUSEamp (mock), pUSEamp-Akt1 (WT-Akt), and pUSEamp-IKK α/β (WT-IKK) for 24 h. At the end of the transfection, the medium was aspirated and replaced with fresh culture medium for 24 h. Cells were treated with 50 μM zerumbone for another 24 h before harvesting.

### Statistical analysis

Results are presented as the mean ± standard error of the mean (S.E.M.) from at least 3 independent experiments. One-way analysis of variance (ANOVA), followed by Dunnet’s test when appropriate, was used to determine the statistical significance of the difference between the means. A *p* value of less than 0.05 was considered statistically significant.

## Results

### Zerumbone induces GBM cell apoptosis

**Figure 1:**
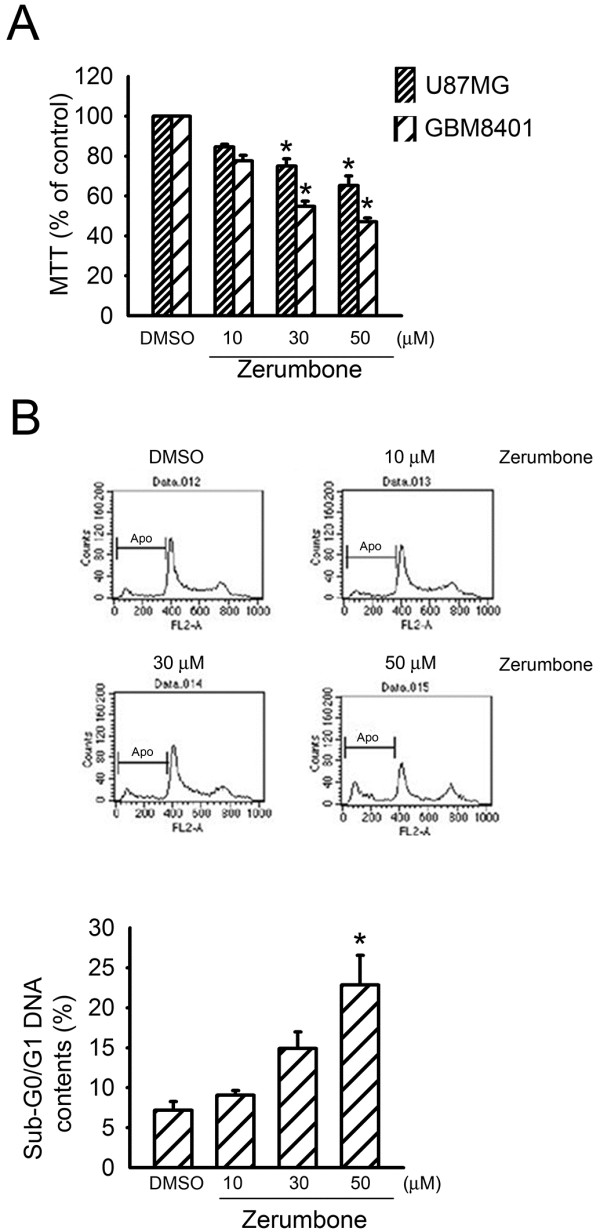
** Zerumbone induced GBM cell death.** (**A**) U87MG and GBM 8401 cells were treated with DMSO or zerumbone at indicated concentrations for 24 h. Cell viability was then determined by the MTT assay. We used GBM8401 cells for further studies, since zerumbone had a greater effect on cell viability in GBM8401 cells. * *p* < 0.05, compared with the control group. (**B**) Cells were treated with DMSO, or zerumbone at indicated concentrations, for 24 h. After treatment, the percentage of sub-G0/G1 contentetric analysis of PI-stained cells as described in Materials and methods. Each column represents the mean ± S.E.M. of at least 3 independent experiments. * *p* < 0.05, compared with the control group.

Treatment of GBM8401 cells with 10, 30, and 50 μM erumbone for 24 h reduced cell viability in a concentration-dependent manner. Zerumbone at the concentration of 30 and 50 μM significantly decreased the viability of GBM8401 cells (up to 45.2 ± 2.5% and 52.9 ± 1.9%, respectively) (n = 3). Zerumbone also decreased cell viability of U87MG cells, another human glioblastoma multiforme cell line. Zerumbone at the concentration of 30 and 50 μM significantly decreased the viability of U87MG cells (up to 26.0 ± 3.6% and 34.8 ± 4.9%, respectively) (n = 3). We used GBM8401 cells for further studies. A flow cytometric analysis of PI-stained cells was then performed to investigate whether zerumbone induces cell death by apoptosis. As shown in Figure 1B, in cells exposed to zerumbone, the percentage of PI-stained cells in the apoptotic region (Apo, sub-G0/G1 peak) increased in in a concentration-dependent manner. The proportion of apoptotic cells increased remarkably from 7.9 ± 1.0% (vehicle-treated control) to 23.9 ± 3.0% after exposure to 50 μM zerumbone.

### Zerumbone triggers caspase activation and PARP cleavage

**Figure 2:**
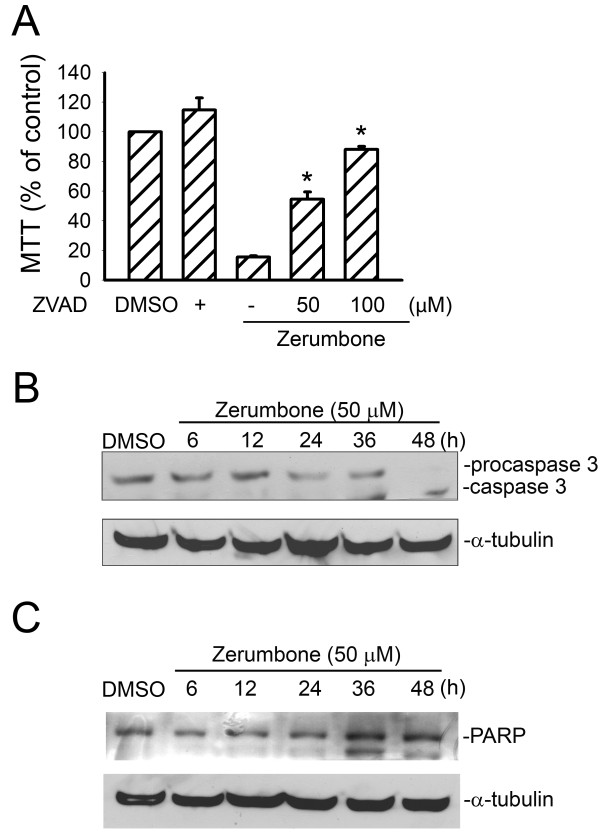
** Zerumbone evoked caspase-3 activation in GBM cells.** (**A**) GBM8401 cells were pretreated with DMSO or zVAD-fmk (50 or 100 μM) for 30 min before the addition of zerumbone (50 μM) for another 24 h. Cell viability was then determined by the MTT assay. *p < 0.05, compared with the group treated with zerumbone alone. Cells were treated with DMSO or zerumbone (50 μM) for indicated time intervals. Protein levels of procaspase-3 and caspase-3 (**B**) and PARP (**C**) were then determined by immunoblotting. Typical traces, representive of data from 3 independent experiments with similar results, are shown.

Caspase-3 has been reported to be downstream of the apoptotic signaling pathway, irrespective of whether intrinsic- or extrinsic signaling mediates the apoptosis [38,39]. Therefore, we sought to determine whether zerumbone-induced GBM8401 cell apoptosis was accompanied by caspase-3 activation. As shown in Figure 2A, zVAD-fmk, a broad-spectrum caspase inhibitor, markedly attenuated the zerumbone-induced decrease in cell viability. Zerumbone (50 μM) induced procaspase-3 degradation and gradual increase of caspase-3 level in GBM cells in a time-dependent manner, within 24 h of exposure to zerumbone (Figure 2B). A selective caspase-3 substrate, PARP, was then used to confirm whether zerumbone-mediated caspase-3 activation resulted in PARP cleavage [38,40,41]. As shown in Figure 2C, zerumbone induced PARP cleavage from a 115- to an 85-kDa fragment. These results suggest that caspase-3 is involved, at least in part, in zerumbone-induced GBM8401 cell apoptosis. 

### Zerumbone induces IKK inactivation in GBM8401 cell apoptosis

**Figure 3:**
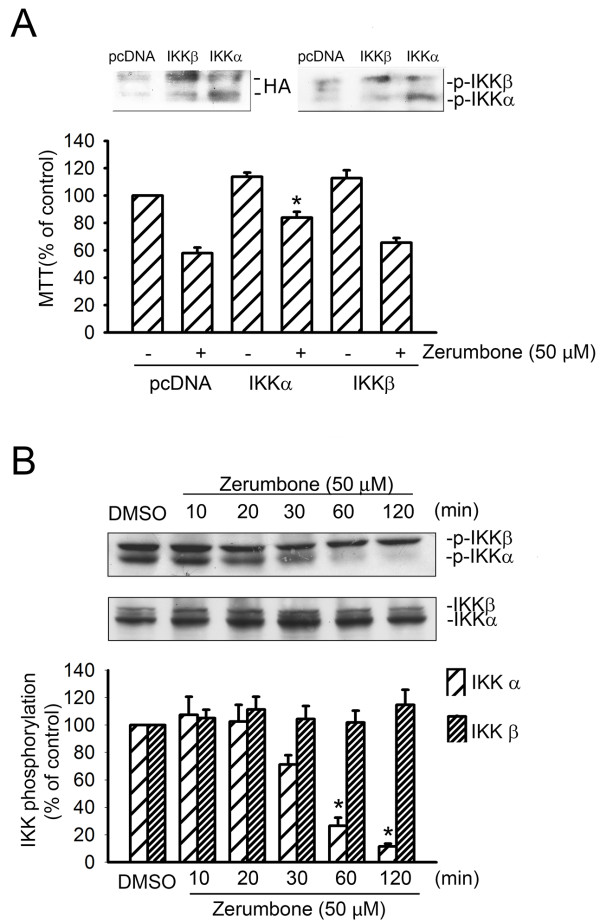
** Zerumbone suppressed IKKα phosphorylation in GBM8401 cells.** (**A**) Cells were transiently transfected with pcDNA (control vector), IKKα or IKKβ and HA for 24 h and then were treated with zerumbone (50 μM) for another 24 h before harvesting. Cell viability was then determined by the MTT assay and western blotting. * *p* < 0.05, compared with the pc DNA-transfected group in the presence of zerumbone. (**B**) Cells were treated with 50 μM zerumbone for the indicated time intervals. IKKα/βphosphorylation was then determined by immunoblotting. Each column represents the mean ± S.E.M. of at least three independent experiments. *p < 0.05, compared with the control group.

Since some recent studies reported that zerumbone inhibits the activation of NFκB and NFκB-related gene expression [5,42]. We then tested whether the IKK- NFκB signaling cascade is involved in zerumbone-induced apoptosis of GBM8401 cells. As shown in Figure 3A, transfection of GBM8401 cells with WT-IKKαrestored the zerumbone-induced decrease in cell viability by 38.7 ± 9.1% (n = 3). However, WT-IKKβ only slightly influenced the effects of zerumbone on the cell viability of GBM 8401 cells. HA level of IKKα and IKKβ both increased after transfection of IKKα and IKKβ. Moreover, transfection of IKKα and IKKβ also augmented phosphorylation level of IKKα and IKKβ respectively. Both of the above documented that IKKα and IKKβ were indeed functional in GBM8401 cells after transfection. In addition, dephosphorylation of both IKKα and IKKβ was observedafter exposure to zerumbone for 60 min (Figure 3B). 

### Akt inactivation is involved in the zerumbone-induced cell apoptosis

**Figure 4:**
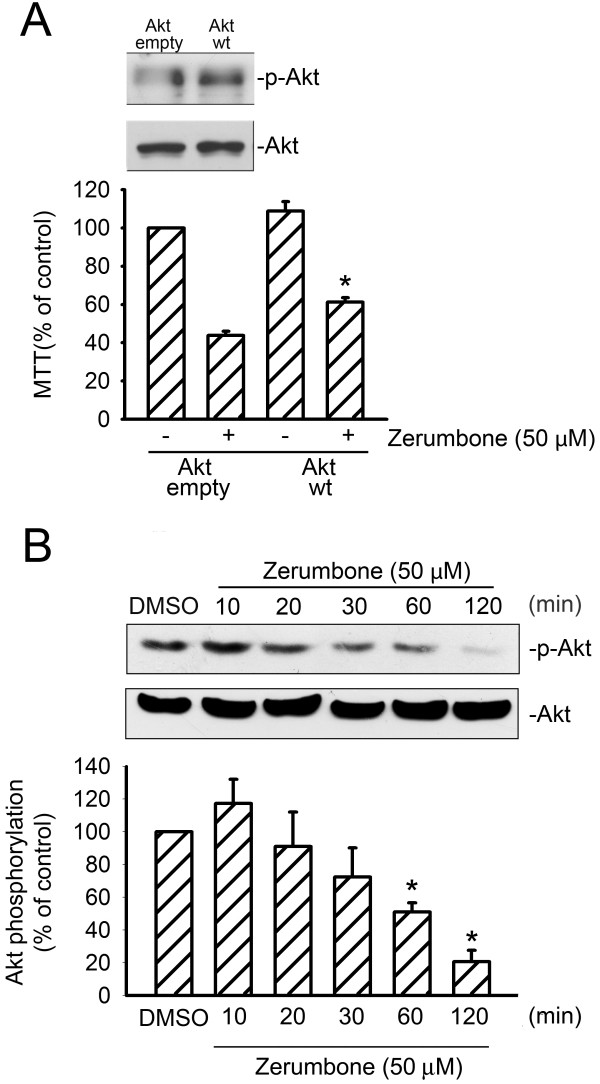
** Akt in zerumbone-induced GBM cell apoptosis.** (**A**) Cells were transfected with empty vector (mock) or wild-type Akt (WT-Akt) for 24 h. Following transfection, cells were treated with vehicle or 50 μM zerumbone for 24 h. Cell viability was then determined by the MTT assay and immunoblotting. Under overexpression of Akt, the phosphorylation level of Akt also increased compared to the mock group, suggesting Akt is functional in GBM8401 cells. Each column represents the mean ± S.E.M. of at least 3 independent experiments. *p < 0.05, compared with the group with trasfected with the empty vector, in the presence of zerumbone. (**B**) Cells were treated with vehicle or zerumbone (50 μM) for indicated time intervals. Phosphorylation status of Akt was then determined by immunoblotting. Each of the columns represents the mean ± S.E.M. of at least three independent experiments. * *p* < 0.05, compared with the control group.

Many studies documented that the PI3K-Akt signaling cascade protects cells from undergoing apoptotic cell death [17,18]. In addition, inhibition of Akt leads to apoptosis in some mammalian cells [22]. To elucidate whether Akt inactivation contributes to zerumbone-induced cell apoptosis, we transfected GBM8401 cells with empty (mock) or WT-Akt prior to zerumbone (50 μM) treatment for 24 h. As shown in Figure 4A, transfection with WT-Akt significantly restored the zerumbone-induced decrease in cell viability. Under overexpression of Akt, Akt phosphorylation level also increased compared to the mock group, suggesting Akt is functional in GBM8401 cells. We then determined whether the extent of Akt phosphorylation is altered by zerumbone. Treatment of cells with zerumbone decreased Akt phosphorylation significantly, as early as 60 min, and this decrease was sustained up to 120 min after zerumbone exposure (Figure 4B). 

### The link between IKK and Akt signaling in zerumbone-induced apoptosis

**Figure 5:**
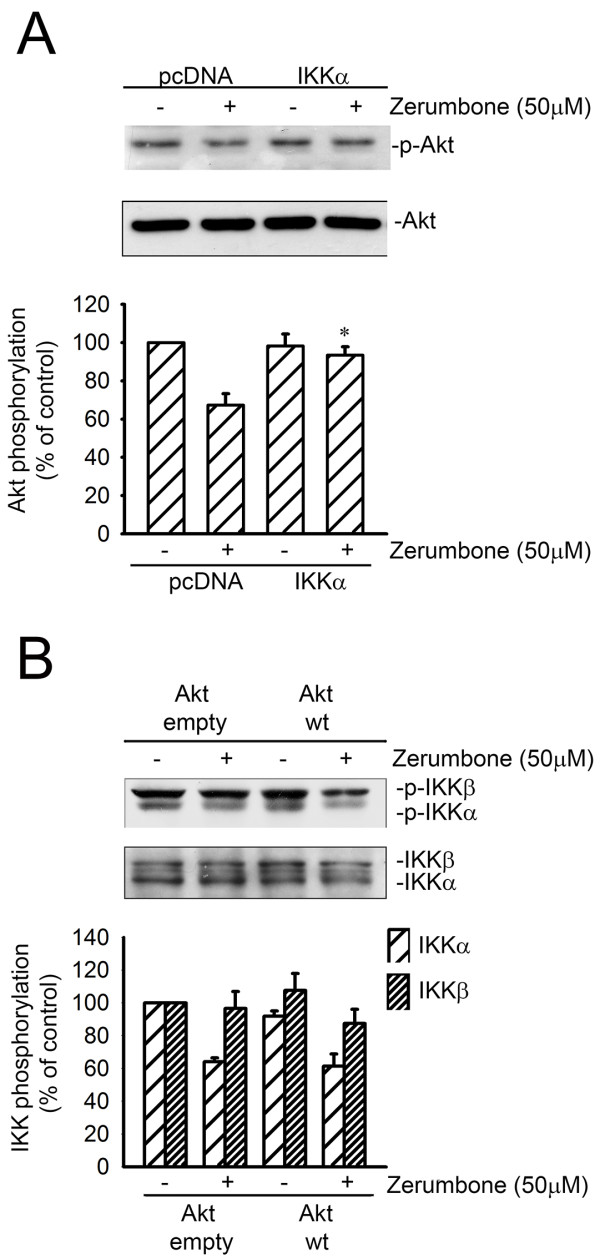
** The link between IKK and Akt in zerumbone-induced apoptosis.** (**A**) Cells were transfected with pcDNA or IKKα forκ 48 h. After transfection, cells were treated with vehicle or 50 μM zerumbone for another 1 h. The phosphorylation of Akt was then determined by immunoblotting. Each column represents the mean ± S.E.M. of at least 3 independent experiments. *p < 0.05, compared with the group trasfected with pcDNA, in the presence of zerumbone. (**B**) GBM cells then were transfected with WT Akt for 48 h. Then the cells were treated with zerumbone for 1 h and the phosphorylation of IKKα and IKKβwas measured by immunoblotting. There was no significant difference of phosphorylation of IKKα and IKKβ between cells transfected with empty vector or with WT Akt before treatment with zerumbone.

To ascertain the link between IKK and Akt signaling downstream of zerumbone, we examined the Akt phosphorylation status in cells transfected with pcDNA (mock) or WT-IKKα in the presence of zerumbone. As shown in Figure 5A, the zerumbone-induced decrease in Akt phosphorylation was significantly restored in cells transfected with WT-IKKα. These results suggest that IKKα may lie upstream of Akt in the apoptotic signaling cascade elicited by zerumbone in GBM8401 cells. In Figure 5B, zerumbone-induced dephosphorylation of IKKα and IKKβ was not reduced remarkably by transfection of GBM cells with WT-Akt. These data suggest that Akt is downstream of IKKα in the zerumbone-induced apoptotic pathway.

### Zerumbone promotes FOXO1 dephophorylation in GBM8401 cell apoptosis

**Figure 6:**
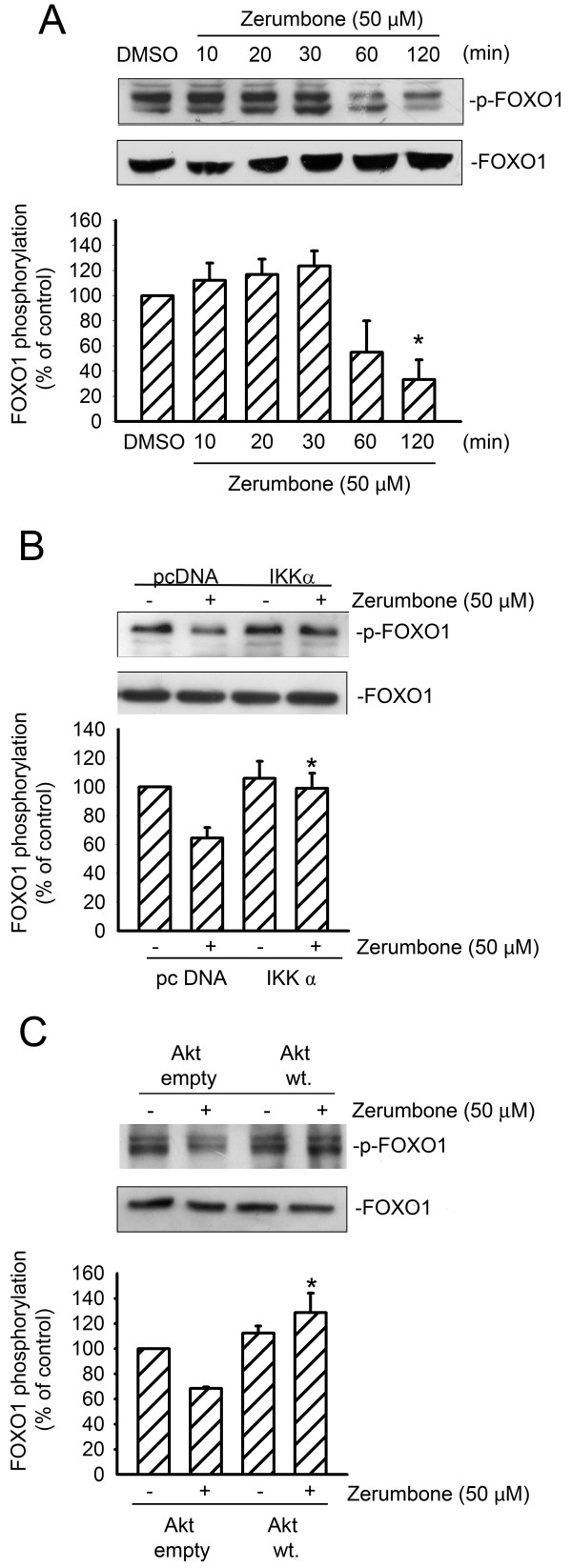
** Zerumbone- induced FOXO1 dephosphorylation in GBM cells.** (**A**) Cells were treated with 50 μM zerumbone for indicated time intervals. FOXO1 phosphorylation status was then evaluated by immunoblotting. Each column represents the mean ± S.E.M. of at least 3 independent experiments. *p <0.05, compared with the control group. (**B**) Cells were transfected with pcDNA or WT IKK for 48 h. After transfection, cells were treated with vehicle or 50 μM zerumbone for 1 h. The phosphorylation status of FOXO1 was then determined by immunoblotting. Each column represents the mean ± S.E.M. of at least three independent experiments. *p < 0.05, compared with the group tranfected with pcDNA, in the presence of zerumbone. (**C**) Cells were transfected with empty vector (mock) and WT Akt. Then cells were treated with vehicle or 50 μM zerumbone and FOXO1 phosphorylation level was measured by immunoblotting. Each column represents the mean ± S.E.M. of at least 3 independent experiments. *p < 0.05, compared with empty vector, in the presence of zerumbone.

We next investigated whether zerumbone-decreased Akt phosphorylation was accompanied by the dephosphorylation of FOXO1, a downstream target of Akt [43]. As shown in Figure 6A, treatment of GBM8401 cells with zerumbone caused FOXO1 dephosphorylation within 120 min. In addition, transfection of cells with WT-IKKα significantly restored the zerumbone-mediated decrease in FOXO1 phosphorylation (Figure 6B). Moreover, as shown in Figure 6C, the phosphorylation of FOXO1 was significantly restored by transfection of GBM cells with WT-Akt. Taken together, these results suggest that FOXO1 takes part in the GBM8401 cells apoptosis induced by zerumbone; and IKKα and Akt both lie upstream of FOXO1 in the apoptotic signaling cascade. 

## Discussion and conclusion

Plant extracts have been used to relieve illness or diseases for several centuries, and anti-cancer properties of specific plant extracts have been the subject of extensive research. Zerumbone, a sesquiterpenoid, is abundant in the rhizomes of the subtropical ginger plant *Zingiber zerumbet* Smith. Some of the dietary terpenoids have exhibited anti-carcinogenic activities in a variety of experiments [44]. Zerumbone was reported to inhibit the proliferation of colon [2] and breast cancers [3], suppress skin tumors in mice [36], and block TNF-induced NF-κB activation in H1299 (lung adenocarcinoma), KBM-5 (human myeloid), A293 (human embryonic kidney), and FaDu (human squamous cell carcinoma) cells [5]. In this study, we demonstrated for the first time that zerumbone can induce human GBM cell apoptosis via inhibition of the IKKα-Akt-FOXO1cascade.

Zerumbone was shown to inhibit TNF- induced NF-κB and IKK activation, and NF-κB- dependent reporter gene expression, in a previous study [5]. In most circumstances, IKK activation triggers phosphorylation, ubiquitination, and degradation of IκB, and then induces nuclear translocation of NF-κB and modification of transcription. However, in our study, overexpression of IKKα suppressed the inactivation of Akt and the dephosphorylation of FOXO1. IKK was also shown previously to phosphorylate FOXO members and induce proteolysis of FOXO members via the ubiquitin-dependent proteasome pathway [29]. Zerumbone may induce apoptosis of GBM cells via an alternative pathway, through the IKK-FOXO cascade. One possible mechanism we cannot rule out is that when NF-κB is overexpressed in GBM cells, phosphorylation of IκB by IKK is inhibited, and abundant IKK may cause phosphorylation and degradation of FOXO1. The link between NF-κB and FOXO1-mediated cell death pathways downstream of IKKα remains to be established. Peng et al. have demonstrated that the FOXO3 protein can suppress NF-κB, either directly or indirectly, by regulating the expression of IκBβ and IκBε proteins [45]. Lee et al. reported that the activation of FOXO3a can induce the expression of κB-ras1, a potent inhibitor of NF-κB signaling, and inhibit the NF-κB pathway [46].

Even though the activation of IKKα and IKKβ mainly initiates NF-κB-mediated transcriptional activation, both IKKα and IKKβ have recently been reported to function independently of each other [29,47]. A number of studies have reported that the Akt kinase activates IKKα rather than IKKβ, especially by phosphorylating the Thr23 residue in IKKα [27,48,49]. These observations explain, at least in part, why zerumbone decreased only IKKα phosphorylation, and the apoptotic actions of zerumbone were restored only in cells transfected with IKKα. The signaling events before IKKα dephosphorylation have not been delineated, but they are likely to involve zerumbone-mediated activation of protein phosphatase or nuclear factor κB- inducing kinase (NIK). Additional studies are needed to characterize the apoptotic signaling cascade triggered by zerumbone, including the involvement of selective protein phosphatases or NIK in zerumbone-induced IKKα dephosphorylation and GBM cell apoptosis.

FOXO members are a group of tumor suppressor proteins with the ability to arrest the cell cycle and to promote apoptosis of tumor cells. Akt can phosphorylate FOXO members, resulting in nuclear export, cytoplasmic retention, and inhibition of transcriptional activity of FOXOs. In this study, we found that IKKα mediates zerumbone-induced decrease in Akt and FOXO1 phosphorylation. These findings suggest that zerumbone may decrease FOXO1 phosphorylation via at least 2 different mechanisms: one, through IKKα-Akt signaling and another, through IKKα directly. The mechanisms by which zerumbone mediates dephosphorylation of FOXO1 remain to be elucidated.

With the balance of the anti- and pro-apoptotic members arbitrating life-or-death decisions, Bcl-2 family proteins may regulate mitochondria-dependent apoptosis [50,51]. Activated Bad, an essential initiator of the apoptotic cascade, is able to form heterodimers with the anti-apoptotic mitochondrial proteins, Bcl-2 and Bcl-xL, to antagonize their antiapoptotic activity and promote the proapoptotic activity of Bax [52,53]. In our study, however, zerumbone did not significantly alter Bcl-2, Bax, or Bcl-xL levels in GBM cells (data not shown). Further investigation may be needed to clarify whether zerumbone affects other Bcl-2 family members such as BH3-only proteins, leading to cell apoptosis in GBM8401 cells.

The half maximal inhibitory concentration (IC50) is the concentration of a compound needed to inhibit a given biological process by half. It is commonly used as a measure of antagonist drug potency in pharmacological research. We calculated the IC50 of zerumbone in GBM8401 and U87MG cells were 47.24 μM and 71.92 μM respectively. Moreover, we reviewed the reported IC50 in other types of cancer cells: colon cancer cells (HT-29): 9.83 μM, breast cancer cells (MCF-7): 10.13 μM [3], cervix cancer cells (HeLa): 20.30 μM [54], and liver cancer cells (Hep G2): 3.45 μM [55]. Among these IC50 of cancer cells, the IC50s of GBM cells (including U87MG and GBM 8401 cells) are higher than cervix and colon cancer cells, and the IC50 of liver cancer cells is relatively low. GBM cells seem more difficultly to be killed than other different kinds of cancer cells. Some people may be worried how to reach such a high level of drugs in brain with contact blood-brain-barrier (BBB). However, there may be some new local delivery methods able to solve the problem, such as biodegradable wafers, convection-enhanced delivery. Other local delivery methods under investigation for malignant gliomas include intracavity administration of radioiodinated TM-601, stereotactic radiotherapy, gene therapy, and tumor-associated radiolabled monoclonal antibodies [56].

The treatment of GBM includes surgery, radiotherapy and adjuvant chemotherapy, Temozolomide is the most update and efficient adjuvant chemotherapy, and the addition of temozolomide improved the median, 2- and 5- year survival significantly compared to radiotherapy alone. Nevertheless, temozolomide can only prolong the median survival of glioblastoma to 14.6 months [35]. Zerumbone can induce dephosphorylation of IKKα, then via Akt dephosphorylation or not, decrease phosphorylation of FOXO1, causing nuclear transport and enhancing transcriptional activity of FOXO1 and triggering GBM cell apoptosis. Therefore, we infer that zerumbone may treat GBM by way of inhibiting its apoptosis resistance.

**Figure 7:**
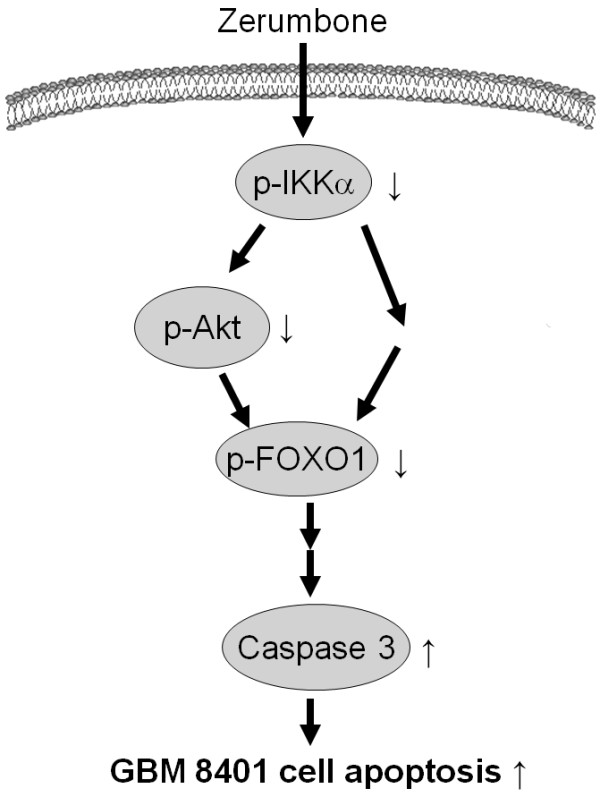
** Schematic summary of apoptotic pathway invoked in zerumbone-induced apoptosis of GBM8401 cell.** Zerumbone-induced inactivation of IKKα leads to FOXO1 dephosphorylation, via Akt dephosphorylation or not, then causing caspase-3 activation, and subsequent cell apoptosis.

In conclusion, the results from this study demonstrated for the first time that zerumbone induces apoptosis of GBM cells by suppressing the IKKα-Akt-FKHR signaling cascade (Figure 7).

## Abbreviations

BAD: Bcl-2-associated death promoter; FKHR: Forkhead in rhabdomyosarcoma; FOXO: Forkhead box, class O; GBM: Glioblastoma multiforme; IKK: IκB kinase; NF-κB: Nuclear factor kappa-light-chain-enhancer of activated B cells; PARP: Poly(ADP-ribose)polymerase; PBS: Phosphate-buffered saline; PI: Propidium iodide; PI3K: Phosphoinositide-3-OH-kinase zVAD-fmk, *N*-benzyloxycarbonyl -Val-Ala-Asp- fluoromethylketone.

## Competing interests

The authors declare no competing interests.

## Authors’ contributions

HYW and MJH designed the study, conducted the experiments, and prepared the manuscript. CCW, BCC, CYH, and MCH provided conceptual suggestions for the study and manuscript preparation; CHL and WTC designed the study, conducted the experiments, and prepared, critically reviewed and submitted the manuscript. All authors read and approved the final manuscript.
